# Breaking the Specific Capacity Limit: 409% Boost in Manganese-Based Redox Flow Batteries at High Current Densities with a MnO_2_ **Semi-Solid** Slurry Electrolyte

**DOI:** 10.1007/s40820-026-02349-8

**Published:** 2026-08-29

**Authors:** Xin Liu, Zhang Chen, Daiqi Zhou, Qi Zhao, Haitao Feng, Yuanyuan Cui, Changsheng Ding, Yanfeng Gao

**Affiliations:** 1 School of Materials Science and Engineering, Shanghai University, Shanghai, 200444 People’s Republic of China; 2 State Key Laboratory of Advanced Refractories, Shanghai University, Shanghai, 200444 People’s Republic of China; 3 Key Laboratory of Comprehensive and Highly Efficient Utilization of Salt Lake Resources, Qinghai Institute of Salt Lakes, Chinese Academy of Sciences, Xining, 810008 People’s Republic of China

**Keywords:** Energy storage, Redox flow battery, Manganese, Semi-solid slurry, Specific capacity

## Abstract

The common-ion effect of SO_4_^2−^ limits the solubility of MnSO_4_ to ≤ 1 M in conventional H_2_SO_4_ electrolytes, resulting in low specific capacity of manganese (Mn)-based redox flow batteries (RFBs). Although MnO_2_ semi-solid RFBs are expected to improve the specific capacity, the sluggish electrochemical reaction kinetics leads to low operating current densities (≤ 1 mA cm^−2^). Herein, we employ a reverse-design strategy by introducing additional high-concentration MnO_2_ into the conventional Mn-based electrolyte. Leveraging the MnO_2_/Mn^2+^ electrochemical reaction successfully circumvents the common-ion effect, raising the concentration of soluble Mn species in the electrolyte to 3.76 M and achieving a specific capacity of 156.2 Ah ${\mathrm{L}}_{{Catholyte}}^{{ - 1}}$ —a 409% increment over reported MnSO_4_-based electrolytes. Subsequently, the reverse disproportionation of electrolytic MnO_2_ generated during the second charging cycle shifts the redox mechanism from the MnO_2_(s)/Mn^2+^ couple to the solution-phase Mn^3+^(aq)/Mn^2+^ pair. This transformation not only elevates the discharge voltage but also enables stable operation at 30 mA cm^−2^, representing a current density 30-fold higher than reported Mn-based semi-solid RFBs. This work demonstrates a rational design strategy for semi-solid slurry electrolytes to enhance the specific capacity of RFBs, thereby advancing their applicability in grid-scale renewable energy storage.

**Supplementary Information:**

The online version contains supplementary material available at https://doi.org/10.1007/s40820-026-02349-8.

## Highlights

- The MnO_2_ /Mn^2+^ electrochemical reaction circumvents the common ion effect, raising the Mn species concentration to 3.76 M and breaking the specific capacity limit of manganese-based redox flow batteries (156.2 Ah $\mathbf{L}_{{Catholyte}}^{{ - 1}})$.
- The reverse disproportionation of electrolytic MnO_2_ shifts the electrochemical mechanism to the solution phase Mn^3+^(aq)/Mn^2+^ redox pair, breaking through the 30 mA cm^−2^ operating current density limit of MnO_2_ based SSFBs.

**Supplementary Information:**

The online version contains supplementary material available at https://doi.org/10.1007/s40820-026-02349-8.

## Graphical abstract

**Figure:**
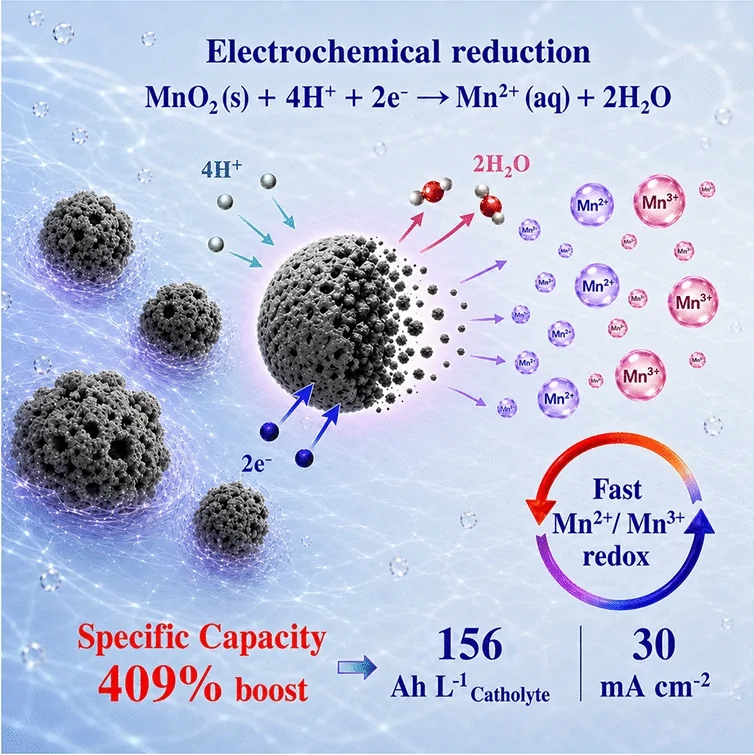

## Introduction

Redox flow batteries (RFBs) offer compelling advantages for renewable energy integration, including long cycle life, inherent safety, and high power density [1, 2]. However, their widespread deployment is constrained by relatively high cost and limited energy density [3–5]. Manganese (Mn)-based RFBs have attracted significant attention due to their high potential (Mn^2+^/Mn^3+^: 1.58 V vs. SHE), cost-effective, and environmental friendliness [6–8]. However, Mn-based RFBs are affected by the spontaneous disproportionation of Mn^3+^, which generates MnO_2_ deposition, leading to the blockage of active reaction sites on the electrode and thus restricting the areal capacity (≈1 mAh cm^−2^) [9–11]. Furthermore, due to the common-ion effect of sulfate ions, the solubility of manganese sulfate monohydrate (MnSO_4_·H_2_O) in 3 M sulfuric acid (H_2_SO_4_) is limited to 1 M, resulting in a relatively low specific capacity (38 Ah ${\mathrm{L}}_{{Catholyte}}^{{ - 1}}$). Previous studies have predominantly focused on addressing the areal capacity limitations of MnO_2_, while strategies for enhancing its specific capacity have received relatively limited attention [12–15].

The electrolyte, comprising active species and supporting electrolyte, serves as a critical component in RFBs, directly governing the specific capacity [16–18]. Attaining high specific capacity requires the concentration of active species to approach the saturation and solubility limits. However, unlike other RFBs system, substituting conventional sulfate-based electrolytes with low-cost alternatives such as MnCl_2_ or HCl to alleviate the SO_4_^2−^ common-ion effect is impractical, since the lower redox potential of Cl^−^/Cl_2_ (1.36 V vs. SHE) relative to Mn^2+^/Mn^3+^ would induce chlorine evolution [19, 20]. To increase the Mn concentration in the electrolyte, MnBr_2_ has been employed as a Mn source, enabling a Mn species concentration of up to 3 M and delivering a specific capacity of 200 Ah ${\mathrm{L}}_{{Catholyte}}^{{ - 1}}$ at a current density of 10 mA cm^−2^ [21]. Furthermore, 3.92 M NaMnO_4_ was utilized as the Mn species in alkaline Mn-based RFBs, achieving a specific capacity of 95 Ah ${\mathrm{L}}_{{Catholyte}}^{{ - 1}}$ at a current density of 30 mA cm^−2^ [22]. Nonetheless, the high material cost of MnBr_2_ and NaMnO_4_ substantially hinders their widespread practical implementation.

Another potential strategy to enhance specific capacity and reduce cost involves leveraging abundant, low-cost solid active materials as semi-solid or suspension electrolytes for RFBs [23–25]. Semi-solid electrolytes incorporating solid–liquid biphasic systems enable a fundamental breakthrough in overcoming the solubility limitations of active materials, achieving ‘infinite solubility’ and thus supporting the development of RFBs with high specific capacity [26, 27]. Recently, various long-cycling semi-solid RFBs have been developed, achieving high specific capacities ranging from 16 to 280 Ah L^−1^ at different current densities [27, 28]. For Mn-based semi-solid RFBs, low-cost MnO_2_/carbon black [29] and Mn_3_O_4_@C/multi-walled carbon nanotube [30] electrolytes have been developed in Mn-based RFBs, achieving specific capacities of 92 and 25 Ah ${\mathrm{L}}_{{Catholyte}}^{{ - 1}}$, respectively. Nevertheless, despite the incorporation of conductive additives such as carbon black and multi-walled carbon nanotubes, the sluggish solid-phase reaction kinetics continue to constrain the electrochemical reaction rate, resulting in low operating current densities (≤ 1 mA cm^−2^). Therefore, it is essential to develop novel strategies to effectively enhance the specific capacity of Mn-based RFBs, while enabling stable operation under high current densities.

**Fig. 1:**
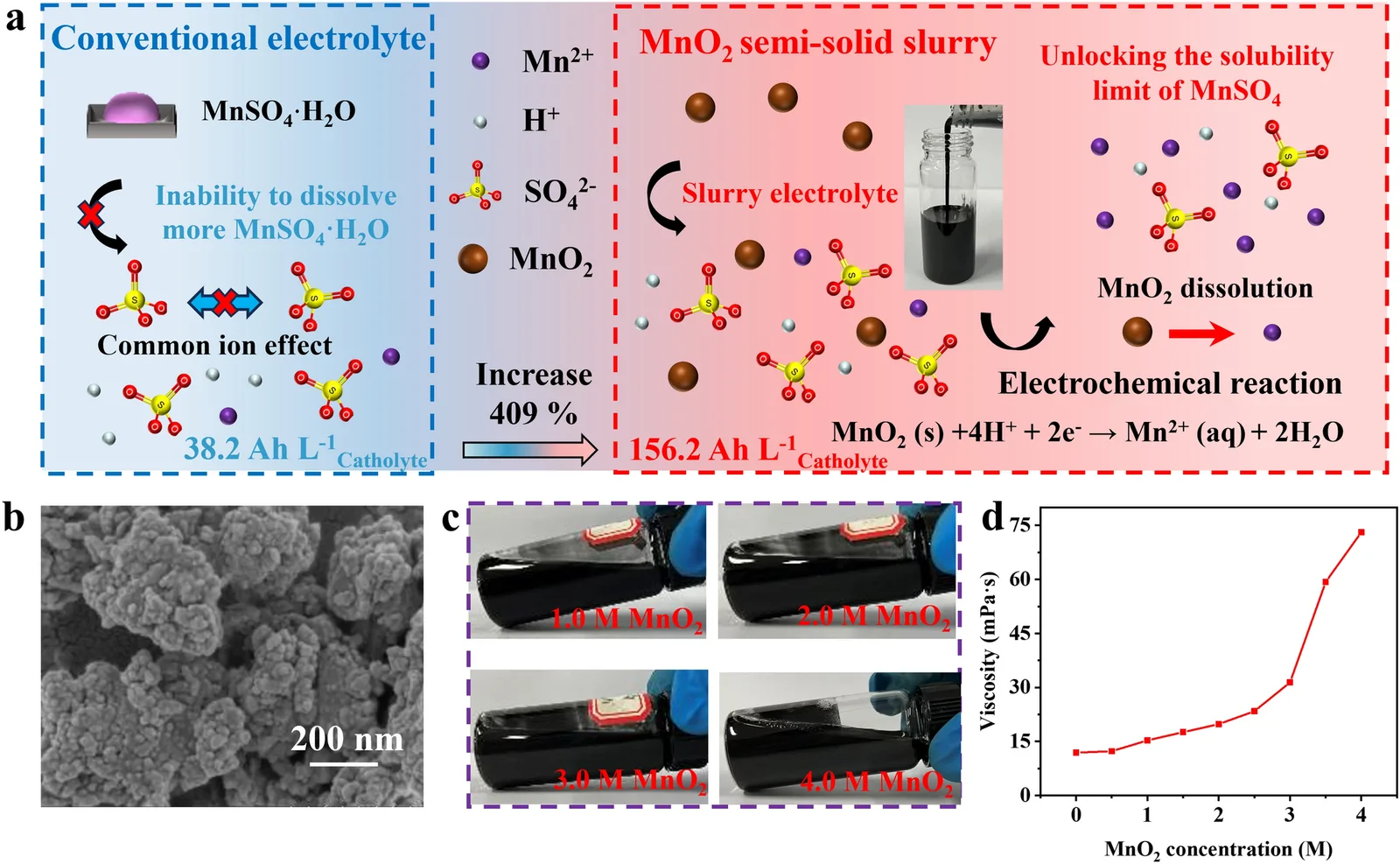
**a** Schematic illustration of the configuration of the MnO_2_ semi-solid slurry RFBs. **b** SEM image of commercial MnO_2_ particles. **c** Optical photographs of 1.0 M, 2.0 M, 3.0 M and 4.0 M MnO_2_ semi-solid slurry electrolytes. **d** Viscosity of slurry electrolytes with different concentrations of MnO_2_ at a shear rate of 60 s^−1^

In conventional Mn-based electrolytes, the low concentration of Mn^2+^ leads to a limited formation of MnO_2_ particles via the disproportionation of Mn^3+^ after charging. Herein, we employ a reverse design strategy by introducing additional high-concentration MnO_2_ particles into the Mn-based electrolyte to enhance the specific capacity of the battery under high current densities. Figure 1a presents a comparative illustration of a conventional Mn-based electrolyte and an MnO_2_ semi-solid slurry. In the conventional Mn-based electrolyte, the common-ion effect of SO_4_^2−^ limits the concentration of soluble Mn species. In contrast, the MnO_2_ semi-solid slurry circumvents the SO_4_^2−^ common-ion effect via the solid–liquid MnO_2_/Mn^2+^ electrochemical reaction, raising the soluble Mn concentration to 3.76 M and achieving a high specific capacity of 156. Ah ${\mathrm{L}}_{{Catholyte}}^{{ - 1}}$. Simultaneously, the reverse disproportionation reaction of the electrolytic MnO_2_ formed upon charging in the second cycle transforms the electrochemical reaction mechanism into the faster solution-phase Mn^3+^/Mn^2+^ redox process, enabling stable battery operation at 30 mA cm^−2^. Ultimately, the semi-solid slurry electrolyte demonstrated stable long-term cycling performance under high specific capacity conditions. This study proposes an innovative prototype design utilizing semisolid slurry electrolytes to enhance the specific capacity of RFBs, which also holds significant potential for extension to other RFBs systems.

## Experimental Section

### Materials

Ferric sulfate (Fe_2_(SO_4_)_3_, Aladdin, 99%), MnSO_4_·H_2_O (Aladdin, 99%), ferrous sulfate heptahydrate (FeSO_4_·7H_2_O, Aladdin, 99%), H_2_SO_4_ (Sinopharm, AR), sodium diphosphate (Na_4_P_2_O_7_·10H_2_O, DAMAO Chemical Reagent Factory, Tianjin, China, 99%), and MnO_2_ (Canrd, AR) were used without further purification. All electrolytes were prepared with deionized water.

### Electrochemical Measurements

CV measurements were taken on a CHI760E electrochemical workstation (Chenhua, Shanghai) using a conventional three-electrode configuration. The MnO_2_ working electrode was fabricated by homogenizing 0.2 g MnO_2_, 0.025 g carbon black, 0.025 g polyvinylidene fluoride (PVDF, Kynar HSV 900, Arkema, France), and 1.2 mL N-methyl-2-pyrrolidone (NMP), and stirring for 10 min. The resulting slurry was painted onto a glassy carbon electrode and dried at 110 °C for 6 h under vacuum. A saturated calomel electrode (SCE) and a platinum strip served as the reference and counter electrodes, respectively. CV scans were conducted between 0.6 and 1.6 V at sweep rates of 5–100 mV s^−1^. EIS was measured at open-circuit potential with a 5 mV amplitude over a frequency range of 0.01 Hz to 100 kHz.

### Battery Test

The flow cell was assembled using graphite felts with geometric areas of 4 or 25 cm^2^ as both positive and negative electrodes. A Nafion 212 membrane was employed to separate the half-cells and mitigate ion crossover. The carbon felt was pressed to 3 mm of the same thickness of the PTFE frame during operation. The electrolyte composition of the catholyte is 1 M of MnSO_4_·H_2_O, 0.5 M of Fe_2_(SO_4_)_3_, 0.1 M Na_4_P_2_O_7_·10H_2_O,and 3 M of H_2_SO_4_. The above-mentioned electrolyte, after the addition of MnO_2_, was first subjected to ultrasonication for 30 min and then magnetically stirred for 10 h to obtain the MnO_2_ semi-solid slurry electrolyte. The anolyte electrolyte was twice the volume of the catholyte electrolyte, and a certain volume of FeSO_4_·7H_2_O solution (1 M FeSO_4_·7H_2_O in 3 M H_2_SO_4_) was added simultaneously, with 1 M of MnO_2_ corresponding to 20 mL. Furthermore, electrodes with areas of 4 and 25 cm^2^ corresponded to catholyte electrolyte volumes of 15 and 20 mL, respectively. Charge/discharge tests were conducted using a LANHE test system (Wuhan, China), with voltage limits set to 1.1 V (charge) and 0.1 V (discharge). The electrolyte flow rate was maintained at 80 mL min^−1^ throughout all experiments.

### Characterization

The conductivities of the slurry/electrolytes were measured by a conductivity meter (DDS-11A, Yueping, Shanghai) at room temperature. Viscosity measurements of slurry were taken on NDJ-9S viscometer with a shear rate of 60 r min^−1^. Elemental concentrations of Mn and Fe in the electrolyte were quantified by ICP (Agilent 720ES). The H⁺ concentration in the electrolyte was determined by titration with NaOH using phenolphthalein as the indicator. The morphological characteristics of the MnO_2_ powders and electrode were examined using SEM (Hitachi S-4700) and TEM (Jeol JEM-2100F). The collected electrolytic MnO_2_ products were isolated via centrifugation, thoroughly rinsed, dried, and subsequently subjected to electron microscopy analysis. Crystalline structure was characterized by XRD (Ultima IV) with Cu Kα radiation over a 2θ range of 30°–70°. Chemical states were analyzed via XPS on a Thermo Scientific Escalab 250Xi system. The specific surface area of MnO_2_ materials was measured by BET method using N_2_ gas as the adsorbent on a Micromeritics ASAP 2460 analyzer. The UV–Vis spectrum of the electrolyte was detected by TU-1901 Beijing Purkinje. The particle size distribution of MnO_2_ was measured using a zeta potential analyzer (Nano-ZS90).

## Results and Discussion

Figure 1b shows the scanning electron microscopy (SEM) image of the commercial MnO_2_ particles, which exhibits a uniform particle size distribution with an average size of approximately 30 nm. The prepared semi-solid slurry electrolyte forms a homogeneous dispersion after prolonged stirring (Figs. 1c and S1). Optical photographs of the MnO_2_ semi-solid electrolyte show that partial sedimentation of MnO_2_ occurs after 10 h of standing, and almost complete sedimentation is achieved after 24 h (Fig. S2). However, the electrolyte becomes uniformly dispersed again after shaking. Since RFBs inherently operate with flowing electrolytes, and MnO_2_ solid particles dissolve into soluble Mn^2+^ in the liquid phase after discharge, the sedimentation issue of MnO_2_ is effectively mitigated. With increasing MnO_2_ concentration, the viscosity of the semi-solid slurry electrolyte rises from an initial 11.9 to 73.1 mPa s at 4 M MnO_2_, corresponding to a solid content of 34.7 wt% in the electrolyte (Fig. 1d). Notably, this work employs graphite felt as the current collector, thereby eliminating the requirement for conductive additives such as carbon black or carbon nanotubes, resulting in significantly lower slurry viscosity compared to previously reported slurry electrolytes (> 1000 mPa s) [30, 31]. Furthermore, since the concentrations of the supporting electrolyte and soluble redox-active species in the electrolyte remain unchanged, the conductivity of the semi-solid slurry electrolytes shows only minor variation (35–38 mS cm^−1^, Fig. S3) across different MnO_2_ loadings.

**Fig. 2:**
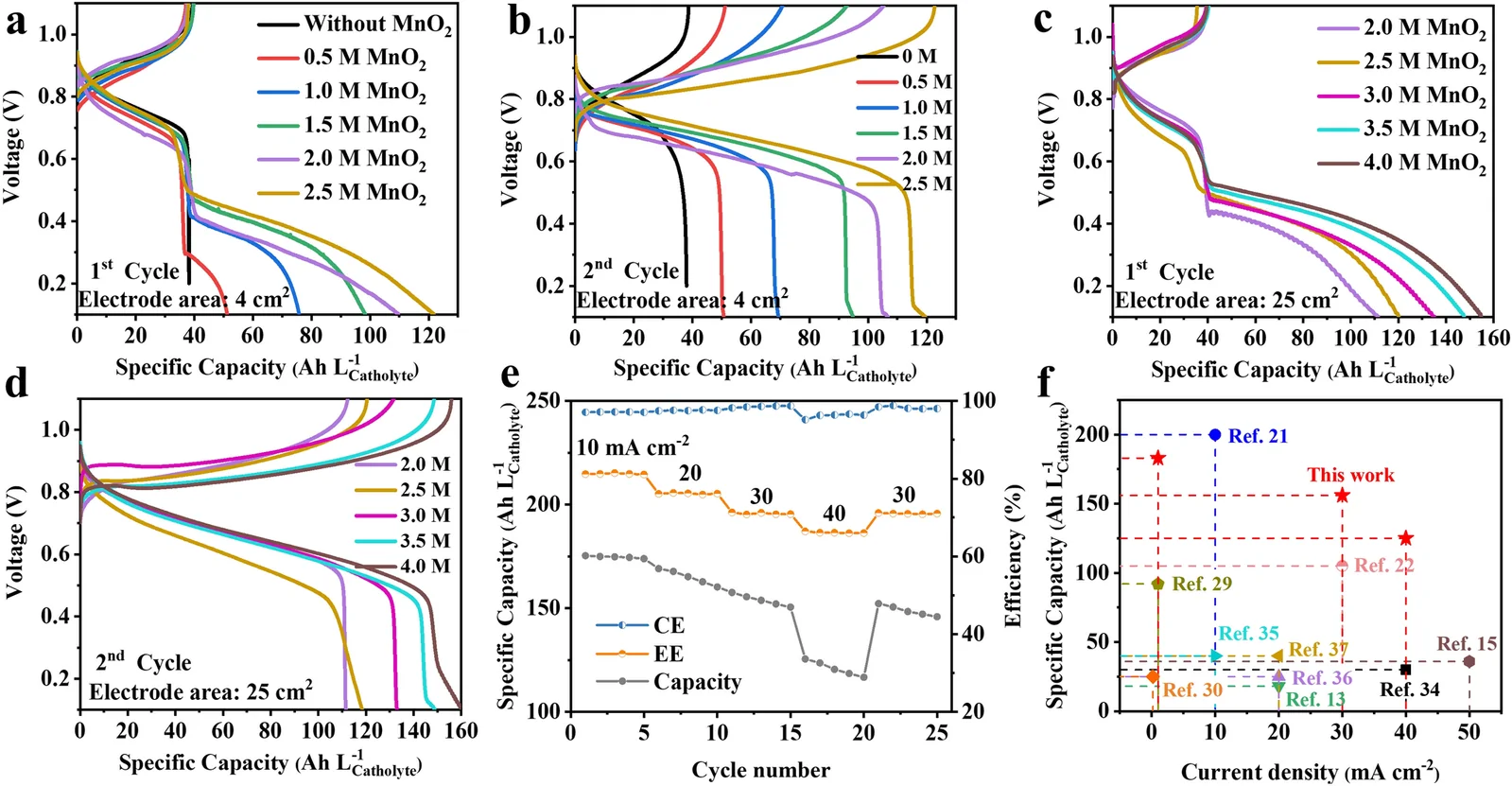
**a** First and **b** second charge–discharge curves of the batteries with different concentrations of MnO_2_ (0 M, 0.5 M, 1.0 M, 1.5 M, 2.0 M, and 2.5 M) at the electrode area of 4 cm^2^. **c** First and **d** second charge–discharge curves of the batteries with different concentrations of MnO_2_ (2.0 M, 2.5 M, 3.0 M, 3.5 M, and 4.0 M) at the electrode area of 25 cm^2^. **e** Rate performance of the semi-solid slurry electrolyte with 4 M MnO_2_ at the electrode area of 25 cm^2^. **f** Comparison of the current density and specific capacity for Mn-based RFBs

Figure 2a displays the first cycle charge–discharge profiles of the semi-solid slurry electrolyte with different MnO_2_ concentrations at a current density of 30 mA cm^−2^ using an electrode area of 4 cm^2^. All electrolytes exhibit an initial charging specific capacity of approximately 38 Ah ${\mathrm{L}}_{{Catholyte}}^{{ - 1}}$, consistent with the concentration of soluble Mn species. In the absence of MnO_2_ in the electrolyte, the discharge specific capacity is only 38 Ah ${\mathrm{L}}_{{Catholyte}}^{{ - 1}}$. Upon the addition of MnO_2_, it is observed that the battery specific capacity increases by 21–24 Ah ${\mathrm{L}}_{{Catholyte}}^{{ - 1}}$ for every 0.5 M increment in MnO_2_ concentration, ultimately reaching 121.8 Ah ${\mathrm{L}}_{{Catholyte}}^{{ - 1}}$ at 2.5 M MnO_2_. In the second cycle (Fig. 2b), the charging specific capacity similarly achieves 123 Ah ${\mathrm{L}}_{{Catholyte}}^{{ - 1}}$ for the 2.5 M MnO_2_ electrolyte, accompanied by the disappearance of the low-voltage plateau and a coulombic efficiency (CE) of 97%. Additionally, the 3 M MnO_2_ slurry electrolyte completes first cycle, achieving a specific capacity of 135 Ah ${\mathrm{L}}_{{Catholyte}}^{{ - 1}}$ (Fig. S4). However, the limited electrode area (the areal capacity reaches 506 mAh cm^−2^) causes cell clogging, resulting in incomplete reaction of MnO_2_ during the second discharge cycle (CE = 39%). To achieve higher specific capacity, electrodes with an enlarged area of 25 cm^2^ were employed to alleviate areal capacity limitations and improve reaction distribution. The charge–discharge performance at elevated MnO_2_ concentrations is shown in Fig. 2c, d. Each 0.5 M increment in MnO_2_ concentration corresponds to a specific capacity increase of 10–14 Ah ${\mathrm{L}}_{{Catholyte}}^{{ - 1}}$, culminating in a notable specific capacity of 156.2 Ah ${\mathrm{L}}_{{Catholyte}}^{{ - 1}}$ at 4 M MnO_2_. Detailed data on specific capacity and areal capacity are summarized in Table S1.

The rate performance of 1 M MnO_2_ semi-solid slurry electrolyte with electrode areas of 4 and 25 cm^2^ is shown in Fig. S4. When the electrode area increases from 4 to 25 cm^2^, the energy efficiency (EE) of the battery improves significantly (79% vs. 75% at 20 mA cm^−2^, 75% vs. 70% at 30 mA cm^−2^, and 69% vs. 63% at 40 mA cm^−2^). The 4 cm^2^ electrode delivered an areal capacity of 284.3 mAh cm^−2^, whereas the 25 cm^2^ electrode only achieved 58.8 mAh cm^−2^. The elevated areal capacity limits mass transport, resulting in deteriorated rate performance. To decouple the effect of areal capacity, Fig. S6 shows the charge–discharge curves of a 4 cm^2^ electrode (areal capacity: 143.4 mAh cm^−2^) and a 25 cm^2^ electrode (areal capacity: 125 mAh cm^−2^) at 30 mA cm^−2^ under different MnO_2_ concentrations. At similar areal capacities, the 4 cm^2^ electrode exhibited a higher EE than the 25 cm^2^ electrode (80.3% vs. 73.1%), suggesting that rate performance depends on both areal capacity and electrolyte concentration. This may be attributed to the fact that higher MnO_2_ concentrations increase the ohmic polarization of the battery, leading to a lower discharge voltage. From experimental observations, the areal capacity is recommended to be maintained below 150 mAh cm^−2^ for electrolyte concentrations < 2 M and below 100 mAh cm^−2^ for concentrations > 2 M. The 4 M MnO_2_ semi-solid slurry electrolyte exhibits an EE of over 71% and a specific capacity above 151 Ah ${\mathrm{L}}_{{Catholyte}}^{{ - 1}}$ at current densities ≤ 30 mA cm^−2^ (Fig. 2e). Notably, a remarkable specific capacity of 183 Ah ${\mathrm{L}}_{{Catholyte}}^{{ - 1}}$ is achieved at 1 mA cm^−2^ (Fig. S7), corresponding to a 17.3% enhancement relative to the value at 30 mA cm^−2^, which stands as the highest record among reported MnO_2_-based semi-solid slurry electrolyte (183 vs. 92 Ah ${\mathrm{L}}_{{Catholyte}}^{{ - 1}}$) [29, 30]. However, the specific capacity fades to 125 Ah ${\mathrm{L}}_{{Catholyte}}^{{ - 1}}$ upon further increasing the current density to 40 mA cm^−2^. At 50 mA cm^−2^, the battery exhibits a sharp decrease in discharge specific capacity with a CE of only 69% (Fig. S8). This is likely due to excessive MnO_2_ in the electrolyte, leading to large ohmic polarization and fast discharge voltage decay.

Based on the model developed by Darling et al. [32, 33], an energy cost analysis of the electrolyte was conducted (see the detailed analysis in Tables S2–S5). The use of low-cost MnO_2_, MnSO_4_, and H_2_SO_4_ as the active materials and supporting electrolyte leads to a substantially lower energy cost for our 4 M MnO_2_ semi-solid slurry electrolyte (33 US$ kWh^−1^) compared to other type RFBs (Fig. S9). Compared with the reported Mn-based RFBs, this study demonstrates superior specific capacity—corresponding to a 409% increment over reported MnSO_4_-based electrolytes, higher current density—reaching 30-fold that of reported Mn-based semi-solid slurry electrolyte (30 vs. 1 mA cm^−2^), lower energy cost (33 vs. 173 US$ kWh^−1^), and greater areal capacity compared to other studies (Fig. 2f and Table S6) [13, 15, 21, 22, 29, 30, 34–37]. Moreover, most reported semi-solid RFBs only deliver a current density of 1 mA cm^−2^, with merely a few reaching 20 mA cm^−2^, whereas this work enables operation at 30–40 mA cm^−2^ while exhibiting an outstanding specific capacity (Fig. S10 and Table S7) [29, 30, 38–47].

**Fig. 3:**
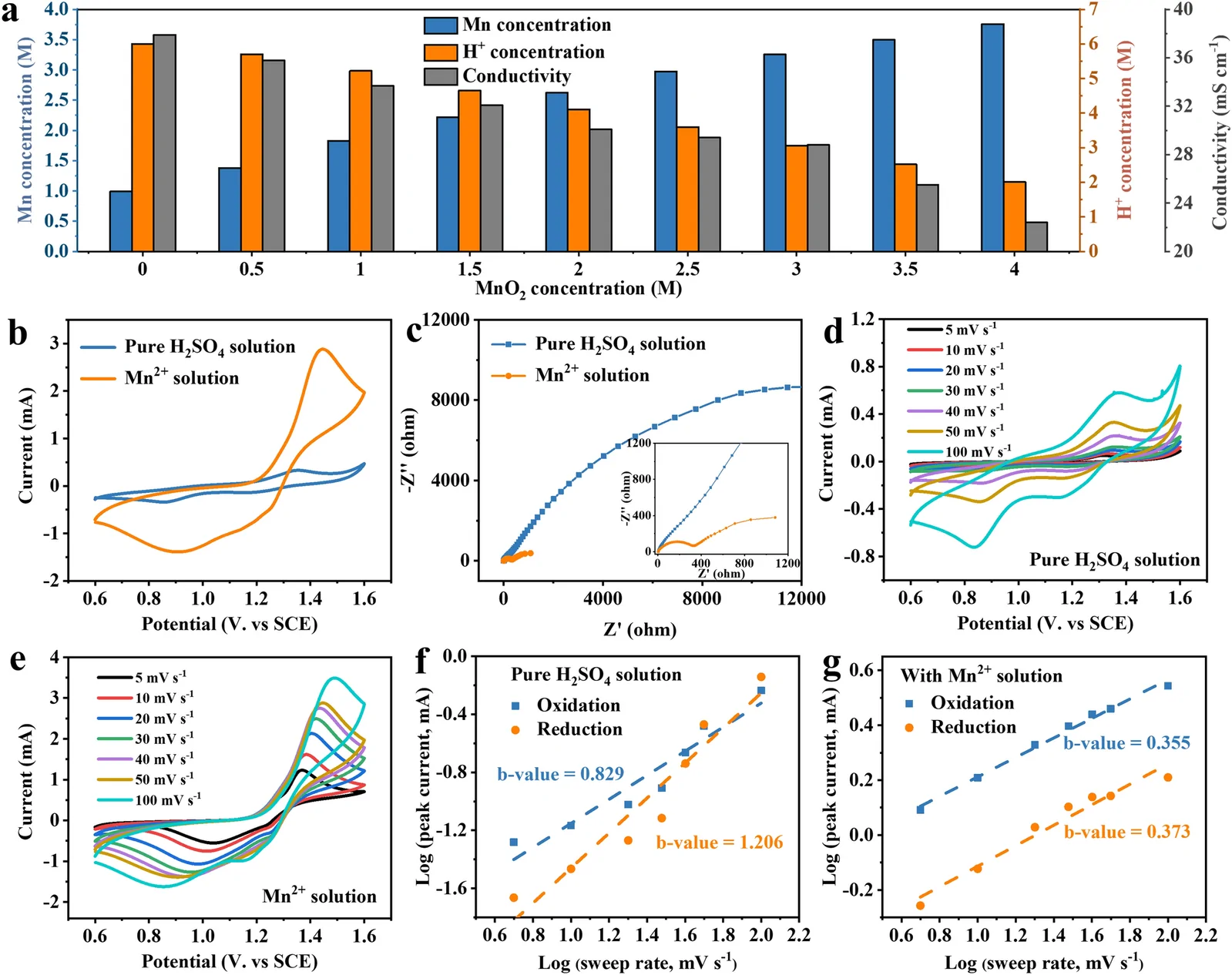
**a** Concentrations of Mn species and H⁺, along with conductivity in the electrolyte after the second cycle at different MnO_2_ concentrations. **b** CV curves at a scan rate of 50 mV s^−1^ and **c** EIS for the pure H_2_SO_4_ and Mn^2+^ solution. CV results of **d** pure H_2_SO_4_ and **e** Mn^2+^ solution at various scan rates. The relationship between oxidation and reduction peak current and scanning rate at **f** pure H_2_SO_4_ and **g** Mn^2+^ solution

The electrolyte after two cycles was centrifuged to remove unreacted solid particles (Fig. S11), with the resulting Mn species concentration, H^+^ concentration, and conductivity shown in Fig. 3a. A progressive increase in the initial MnO_2_ loading correlates directly with higher soluble Mn concentrations in the post-cycled electrolyte, confirming the feasibility of the proposed mechanism: The electrochemical conversion of solid MnO_2_ to Mn^2+^ at the electrode effectively enhances the concentration of active species in the electrolyte. At MnO_2_ concentrations ≤ 2.0 M, each 0.5 M increment raises the soluble Mn concentration by 0.39–0.45 M, reaching 2.58 M at 2.0 M MnO_2_. Above this concentration, the incremental gain decreases to 0.24–0.35 M, culminating in 3.76 M at 4 M MnO_2_. Overall, the utilization rate of MnO_2_ in the semi-solid slurry system exceeds 69% (Fig. S12). After the completion of the electrolyte reaction, a small amount of MnO_2_ inevitably sediments in the electrolyte reservoir, which accounts for the decreasing utilization efficiency of MnO_2_ at concentrations above 3 M. Furthermore, the electrolyte viscosity decreases after MnO_2_ is converted to soluble Mn^2+^ ions, and the higher the initial MnO_2_ concentration, the more significant the viscosity reduction (the viscosity of the 4 M MnO_2_ electrolyte decreases from 73.1 to 39.4 mPa s) (Fig. S13). For every 0.5 M MnO_2_ consumed, the H⁺ concentration in the catholyte decreases by approximately 0.5 M. To compensate for H⁺ consumption, H⁺ from the anolyte migrates across the membrane and partially replenishes the catholyte. As indicated in Note S1, the larger volume of anolyte ensures sufficient H⁺ supply to sustain the MnO_2_ electrochemistry (Fig. S14). Notably, this decline in H⁺ concentration consequently diminishes the electrolyte conductivity from an initial value of 37.9 to 22.4 mS cm^−1^ at 4 M MnO_2_. Nevertheless, the lowered conductivity does not impair the reversibility of the electrochemical reaction (Fig. S15).

It is hypothesized that the low-voltage plateau (< 0.4 V) observed during the first discharge cycle arises from two distinct electrochemical mechanisms [34, 48, 49]:

1 $${\mathrm{MnO}}_{{2}} \left( {\mathrm{s}} \right) + {\text{ 4H}}^{ + } + {\text{ 2e}}^{ - } \to {\mathrm{Mn}}^{{{2} + }} \left( {{\mathrm{aq}}} \right) \, + {\text{ 2H}}_{{2}} {\mathrm{O}} E = { 1}.{\text{23 V vs}}.{\text{ SHE}}$$

2 $${\mathrm{Mn}}^{{{3} + }} \left( {{\mathrm{aq}}} \right) + {\text{ e}}^{ - } \to {\mathrm{Mn}}^{{{2} + }} \left( {{\mathrm{aq}}} \right) E = { 1}.{\text{56 V vs}}.{\text{ SHE}}$$

To validate this hypothesis, systematic electrochemical characterization was performed by painting commercial MnO_2_ slurry onto a glassy carbon electrode and evaluating its behavior in both pure H_2_SO_4_ solution and Mn^2+^-containing electrolyte. In the Mn^2+^-containing electrolyte, the Mn^3+^/Mn^2+^ redox reaction (Eq. 2) dominates the electrochemical process, whereas in pure H_2_SO_4_ solution, the MnO_2_/Mn^2+^ reaction (Eq. 1) becomes operative, resulting in a 50 mV negative shift in the cyclic voltammetry (CV) curve (Fig. 3b). Concurrently, electrochemical impedance spectroscopy (EIS) reveals a significantly higher charge transfer resistance for the MnO_2_/Mn^2+^ reaction in pure H_2_SO_4_ solution (Fig. 3c). The MnO_2_ in Mn^2+^-containing electrolyte demonstrates superior CV stability compared to that pure H_2_SO_4_ solution across 5–100 mV s^−1^ (Fig. 3d, e). Furthermore, the b-value derived from the relationship between reduction peak current and scan rate (Fig. 3f, g) indicates that the MnO_2_/Mn^2+^ reaction is primarily surface-controlled capacitive behavior (b = 1.206, close to 1), while the Mn^3+^/Mn^2+^ reaction demonstrates diffusion behavior (b = 0.373, close to 0.5), which may account for the observed variation in EIS [50–52]. In summary, after Mn^3+^ is depleted during the discharge process, the MnO_2_ deposited on the graphite felt (Fig. S16) is converted to Mn^2+^ via the reaction described in Eq. 1. The combined effect of reduced potential and elevated charge transfer resistance results in the emergence of the low-voltage plateau.

**Fig. 4:**
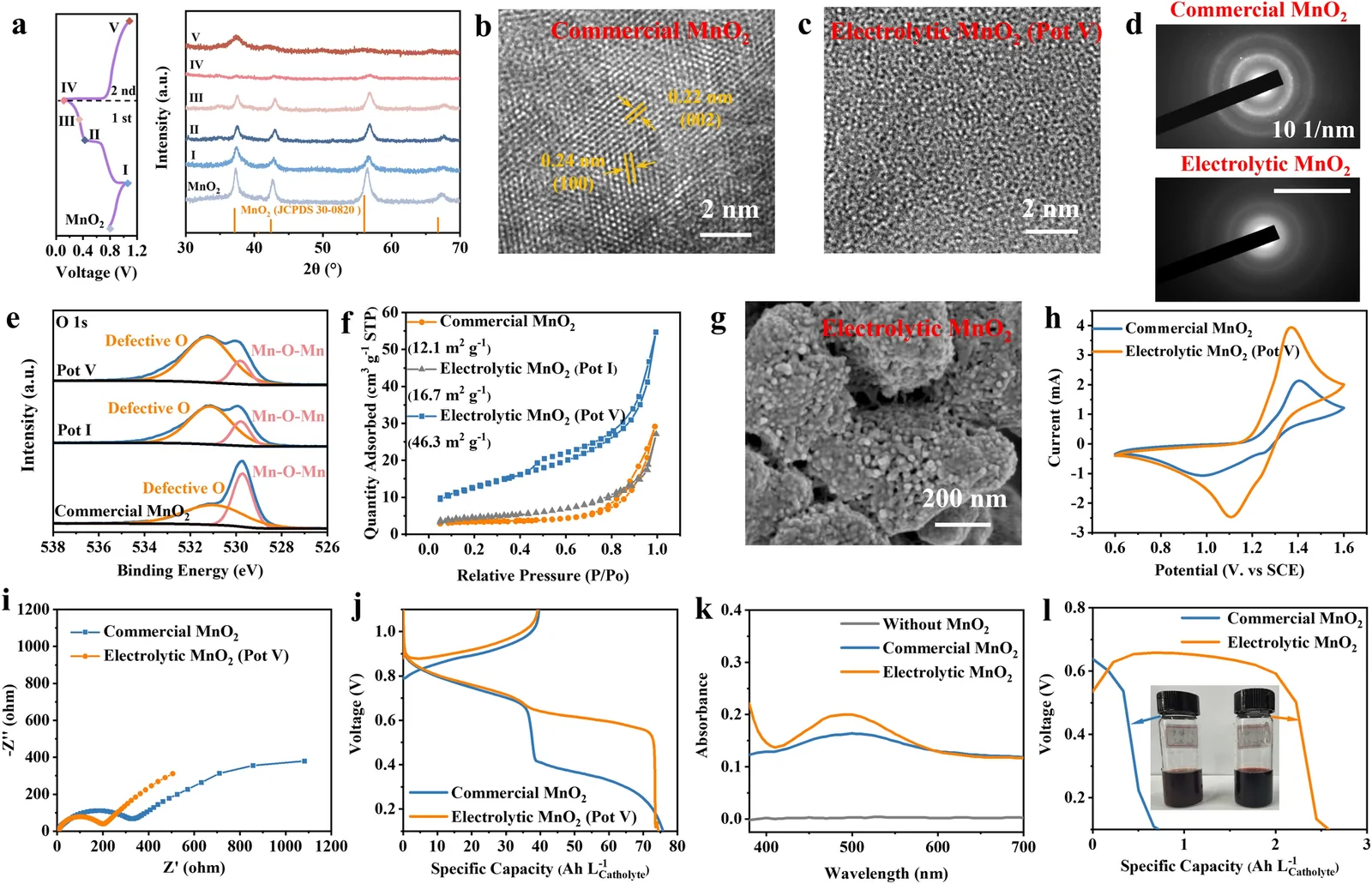
**a** XRD characterization of solid particles in the electrolyte at different charge/discharge stages. TEM images of the **b** commercial MnO_2_ and **c** electrolytic MnO_2_ (Pot Ⅴ, after full charging in the second cycle). **d** SAED patterns of the commercial and electrolytic MnO_2_. **e** XPS spectra of O 1*s* and **f** Nitrogen adsorption/desorption isothermal curves of MnO_2_ at different stages (commercial MnO_2_, Pot Ⅰ and Pot Ⅴ). **g** SEM images of electrolytic MnO_2_ (Pot Ⅴ) particles. **h** CV curves at a scan rate of 20 mV s^−1^, **i** EIS and **j** the first charge–discharge curves of 1 M commercial MnO_2_ and electrolytic MnO_2_ (Pot Ⅴ). **k** UV–Vis spectra of the different electrolytes. **l** Discharge curves of electrolytes after 2 h stirring of commercial/electrolytic MnO_2_ in pristine electrolyte and solid separation (Inset: digital photos of electrolytes)

To elucidate the structural evolution of MnO_2_ during cycling, X-ray diffraction (XRD) patterns of solid particles at different charge/discharge stages were analyzed (Fig. 4a). All samples exhibit characteristic diffraction peaks of MnO_2_ (a hexagonal phase MnO_2_, JCPDS 30–0820) [53], with a notable peak broadening observed in the fully charged state of the second cycle (Pot V, electrolytic MnO_2_). Combined with transmission electron microscope (TEM) morphology analysis, it reveals that the commercial MnO_2_ transforms from an ordered crystalline structure with clear lattice fringes (0.22 and 0.24 nm) into a disordered amorphous phase (Pot V) (Fig. 4b, c). Furthermore, the MnO_2_ obtained after the first charging cycle (Pot I) display a mixed composition of both crystalline and amorphous MnO_2_ (Fig. S17). Selected area electron diffraction (SAED) patterns reveal that both MnO_2_ samples show polycrystalline concentric rings, with commercial MnO_2_ exhibiting sharp diffraction spots and electrolytic MnO_2_ showing continuous diffuse halos (Fig. 4d). Besides, The O 1*s* X-ray photoelectron spectrum (XPS) spectrum was fitted into two peaks at 529.7 eV (Mn–O–Mn) and 531.2 eV (defective O) [54]. The signal for the defective O peak strongly increases (Fig. 4e) in samples Pot Ⅰ and Pot Ⅴ. These findings collectively demonstrate that the crystal structure of MnO_2_ undergoes transformation after the second charging cycle, corroborating previously reported results [15, 55]. Furthermore, electrolytic MnO_2_ (Pot V) exhibits reduced particle size (44.5 vs. 50.9 nm, Fig. S18) with a more porous structure (Figs. 4g and S19) and a larger specific surface area compared to commercial MnO_2_ (46.3 vs. 12.1 m^2^ g^−1^, Fig. 4f).

To clarify the electrochemical reaction mechanism in the second cycle, electrochemical performance was conducted by painting commercial MnO_2_ and electrolytic MnO_2_ (Pot V) slurries onto a glassy carbon electrode, respectively. The results demonstrate that the electrolytic MnO_2_ exhibits a narrower potential gap (ΔEp = 0.26 vs. 0.42 V for commercial MnO_2_, Fig. 4h) and lower charge transfer resistance (Figs. 4i and S20), and exhibits diffusion-controlled behavior (b = 0.473, Fig. S21, indicating enhanced electrochemical reaction kinetics [56]. To further compare their electrochemical behavior, the two types of 1 M MnO_2_ were separately prepared into semi-solid slurry electrolytes. A distinct red coloration, characteristic of Mn^3+^, is observed in the electrolytic MnO_2_ electrolyte (Fig. S22) [52, 57]. More importantly, Fig. 4j demonstrates that the low-voltage plateau (< 0.4 V) during the first cycle disappears for the electrolytic MnO_2_ electrolyte, with only a single high-voltage plateau (> 0.6 V) remaining, indicating distinct electrochemical reaction pathways during discharge between the commercial MnO_2_ and electrolytic MnO_2_ (Pot V). As demonstrated in our prior work, the Na_4_P_2_O_7_ additive regulates MnO_2_ deposition behavior to enhance battery areal capacity, yet it does not change the charge–discharge mechanism of the resulting MnO_2_ [15]. To verify this, an electrolyte without the Na_4_P_2_O_7_ additive is prepared in Fig. S23. Charge–discharge tests still exhibit a charge–discharge mechanism transition in the second cycle (absence of the low-discharge-voltage plateau).

UV–Vis spectra of the two MnO_2_ electrolytes both exhibit a broad absorption peak centered at approximately 500 nm (the characteristic UV absorption peak of Mn^3+^ [57]), with the electrolytic MnO_2_ sample showing a significantly higher peak intensity (Fig. 4k). In contrast, the electrolyte without MnO_2_ shows little absorbance at these wavelengths. This confirms that both types of MnO_2_ undergo reverse disproportionation reactions, with electrolytic MnO_2_ displaying higher reaction activity. Commercial and electrolytic MnO_2_ were added to the pristine electrolyte, stirred for 2 h, and centrifuged to separate solids from solution. The dried MnO_2_ particles show mass losses of 16.6% (commercial MnO_2_) and 25.2% (electrolytic MnO_2_), and the separated red electrolytes deliver specific capacities of 0.72 and 2.58 Ah ${\mathrm{L}}_{{Catholyte}}^{{ - 1}}$, respectively (Fig. 4l).

Based on the above experimental results, the underlying mechanism for the disappearance of the low-voltage plateau in the second cycle may be explained as follows. During the first cycle, commercial MnO_2_ discharge proceeds via a MnO_2_/Mn^2+^ reaction (Eq. 1), characterized by a lower voltage and slower reaction rates, leading to the emergence of the low-voltage plateau. In contrast, the electrolytic MnO_2_ formed in the subsequent cycle is newly generated through the disproportionation of Mn^3+^. This electrolytic MnO_2_ exhibits an amorphous structure, smaller particle size, larger specific surface area, and enhanced reaction kinetics, favoring the reverse disproportionation reaction to form Mn^3+^ (Mn^2+^ (aq) + MnO_2_ (s) + 4H^+^  → 2Mn^3+^ (aq) + 2H_2_O). The electrochemical reaction shifts to the liquid-phase Mn^3+^/Mn^2+^ couple (Eq. 2), which operates at a higher voltage and exhibits faster reaction kinetics, ultimately leading to the disappearance of the low-voltage plateau in subsequent cycles.

**Fig. 5:**
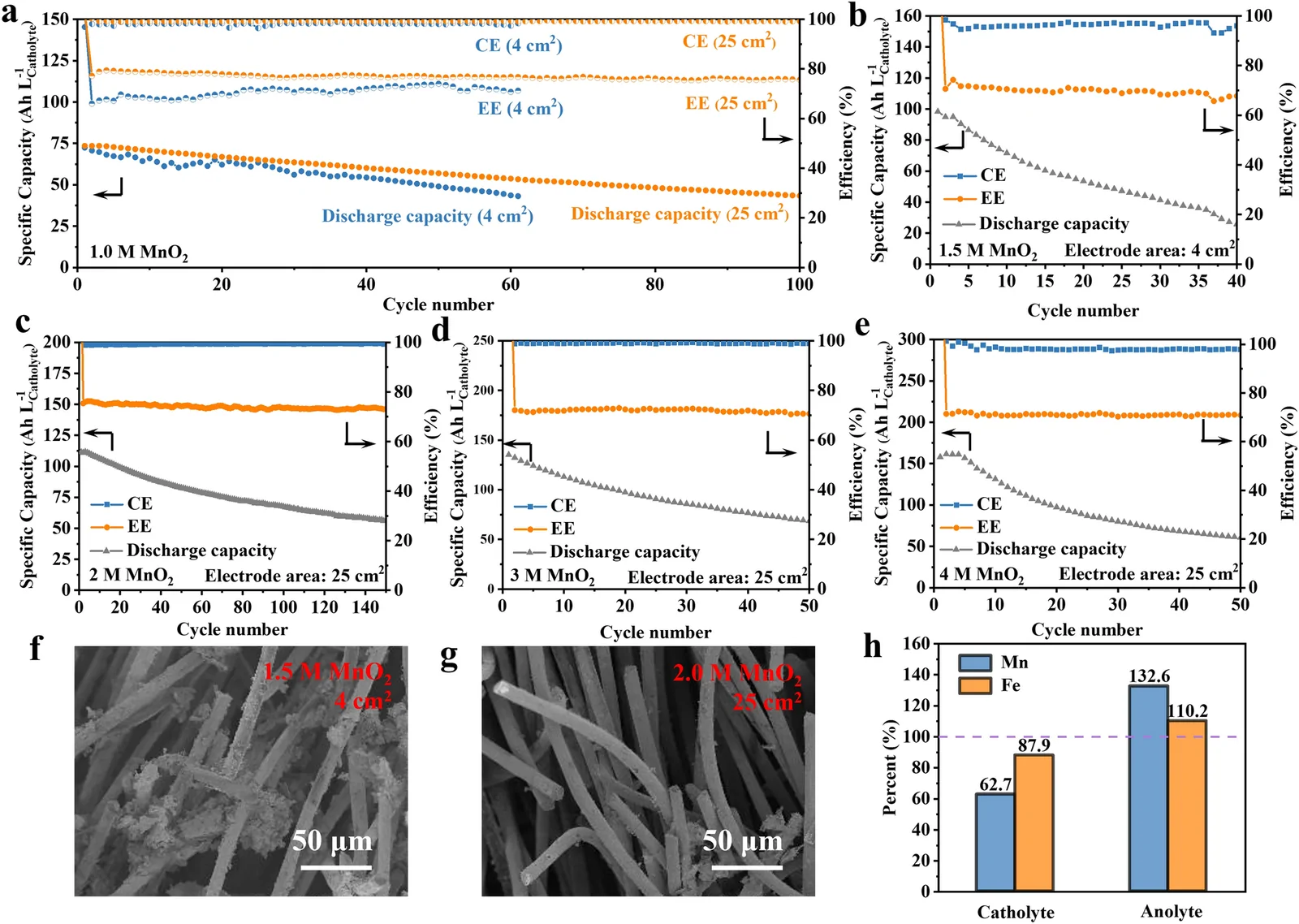
**a** Long-term cycling performance of the electrolyte with 1 M MnO_2_ at the electrode area of 4 and 25 cm^2^. **b** Long-term cycling performance of the electrolyte with 1.5 M MnO_2_ at the electrode area of 4 cm^2^. Long-term cycling performance of the electrolyte with **c** 2.0 M, **d** 3.0 M, and **e** 4.0 M MnO_2_ at the electrode area of 25 cm^2^. The SEM images of electrode after long-term cycling under conditions of **f** 1.5 M MnO_2_ with 4 cm^2^ area and **g** 2.0 M MnO_2_ with 25 cm^2^ electrode area, respectively. **h** Evolution of Mn and Fe concentrations in the catholyte and anolyte over 100 cycles using a 1 M MnO_2_ electrolyte

To verify the reaction stability under extended cycling, long-term cycling tests were conducted on semi-solid slurries with different electrode areas and varying MnO_2_ concentrations. In Fig. 5a, the 1.0 M MnO_2_ electrolyte at current density of 30 mA cm^−2^ using a 25 cm^2^ electrode demonstrates stable performance with a 99% CE and 76% EE, while maintaining 59% capacity after 100 cycles—significantly superior to the battery with a 4 cm^2^ electrode (retained 59% capacity after 60 cycles). Notably, when operating with a 1.5 M MnO_2_ electrolyte at an electrode area of 4 cm^2^, the battery exhibits relatively low CE (96%) and EE (69%), along with rapid capacity decay—retaining only 26% capacity after 40 cycles (Figs. 5b and S24). However, when the electrode area is increased to 25 cm^2^, electrolytes with 2, 3, and 4 M MnO_2_ all achieved high CE (99.3%, 99.0%, and 98.2%, respectively) and EE (73.9%, 71.9%, and 71.1%, respectively), while maintaining capacity retentions of 50% after 150 cycles, 51% after 50 cycles, and 38% after 50 cycles, respectively (Fig. 5c–e). Furthermore, the capacity-voltage profiles in subsequent cycles indicate that after the second cycle, the electrochemical reaction remains stabilized as the Mn^3+^/Mn^2+^ redox process without the appearance of a low-voltage plateau (Fig. S25).

To investigate the cause of capacity degradation, the morphology of the post-cycled electrodes and the active materials concentrations in the electrolyte were examined. When operated with a 1.5 M MnO_2_ electrolyte at an electrode area of 4 cm^2^, the battery requires 575 h to complete 40 cycles and achieves an exceptional areal capacity of 369 mAh cm^−2^—the highest value reported to date in the literature. The excessive areal capacity results in incomplete reaction of MnO_2_, causing its accumulation on the electrode (Fig. 5f), while the extended cycling duration severely exacerbates ion crossover through the membrane (Fig. S26). These issues collectively contribute to the rapid capacity decay observed. When the electrode area is increased to 25 cm^2^, the areal capacities reach 89, 108, and 125 mAh cm^−2^ for the batteries using 2, 3, and 4 M MnO_2_ electrolytes, respectively. These areal capacities are sufficient to ensure complete reaction of MnO_2_, thus preventing detectable particle deposition on the electrode (Figs. 5g and S27). Inductively coupled plasma (ICP) analysis of the 1 M MnO_2_ electrolyte after 100 cycles (Fig. 5h) reveals that the Mn species in the catholyte retains only 62.7% of its second value (decreasing from 1.83 to 1.15 M). This reduction may be attributed to the initial Mn species concentration imbalance between the catholyte and anolyte (1.83 vs. 0.67 M), which promoted Mn ion migration across the membrane, resulting in active material loss and capacity decay. An additional factor contributing to capacity fade may be the elevated concentration of MnO_2_ in the electrolyte, where excessive particle agglomeration results in its retention in the electrolyte without participating in the electrochemical reaction.

Energy density depends on cell voltage and specific capacity, and the anolyte redox couple determines the total cell voltage. In the present configuration, employing the Fe^3+^/Fe^2+^ redox couple (0.77 V vs. SHE) as the anolyte and pairing it with the cathodic Mn^2+^/Mn^3+^ couple (1.56 V vs. SHE) yield an open-circuit voltage of 0.79 V and an energy density of 52.1 Wh ${\mathrm{L}}_{{Catholyte}}^{{ - 1}}$ at 1 M MnO_2_. For comparison, replacing the Fe^3+^/Fe^2+^ anolytes with the V^3+^/V^2+^ redox couple (–0.26 V vs. SHE) results in a twofold increase in the battery's discharge voltage at 30 mA cm^−2^ (1.45 vs. 0.71 V, Fig.  S28). This leads to a corresponding twofold enhancement in discharge energy density (97.1 vs 48.2 Wh ${\mathrm{L}}_{{Catholyte}}^{{ - 1}}$), with stable cycling maintained over 100 cycles. Similarly, employing alternative anolytes such as TiO^2+^/Ti^3+^ (–0.15 V vs. SHE) [12, 58], Cd^2+^/Cd (–0.40 V vs. SHE) [21], or Sn^2+^/Sn (–0.14 V vs. SHE) [59] could deliver higher voltages of approximately 1.71, 1.96, and 1.70 V, respectively, thereby potentially further increasing the energy density of the battery. Our work demonstrates that the semi-solid slurry electrolyte strategy significantly enhances the specific capacity of batteries while enabling operation at higher current densities. Although the issue of long-cycle capacity retention remains to be fully resolved, future studies may focus on balancing the concentrations between the catholyte and anolyte, or developing ion-selective membranes to suppress crossover-induced capacity fade, or employing effective dispersants to mitigate MnO_2_ agglomeration. In addition, strategies similar to those used for Mn-based cathodes in other aqueous batteries, including interface engineering, electrolyte optimization, carbon composite design, and structural engineering, can be adopted to improve battery performance [60–62].

## Conclusions

In summary, the proposed semi-solid slurry electrolyte design overcomes the solubility limitation of Mn species in conventional Mn-based electrolytes. By leveraging the electrochemical reaction of MnO_2_, the concentration of Mn species in the electrolyte was increased to 3.76 M, achieving a high specific capacity of 156.2 Ah ${\mathrm{L}}_{{Catholyte}}^{{ - 1}}$—a 409% increment compared to reported MnSO_4_-based electrolytes. Detailed mechanistic studies reveal that the electrochemical reaction transitions from the MnO_2_(s)/Mn^2+^ redox couple to the solution-phase Mn^3+^(aq)/Mn^2+^ pair, with the system stabilizing after the second cycle. Long-term cycling tests further demonstrate sustained reaction stability. The semi-solid slurry electrolyte concept introduced in this study holds promise for generalization to other RFBs systems, offering a viable pathway toward practical high-energy–density storage. Future efforts should focus on extending the cycle life and pairing the system with lower-potential anodes to improve energy density of battery.

## Supplementary Information

Below is the link to the electronic supplementary material.Supplementary file1 (DOCX 16770 KB)
