# Supplementary material for: Breaking the Specific Capacity Limit: 409% Boost in Manganese-Based Redox Flow Batteries at High Current Densities with a MnO2 Semi-Solid Slurry Electrolyte
Source: Nanomicro Lett. 2026 Aug 29;19:41. doi: 10.1007/s40820-026-02349-8 (PMC13526001; doi:10.1007/s40820-026-02349-8)
Supplement: Supplementary file 1 [file 40820_2026_2349_MOESM1_ESM.docx]

Supporting Information for

**Breaking the Specific Capacity Limit: 409% Boost in Manganese-Based Redox Flow Batteries at High Current Densities with a MnO_2_ Semi-Solid Slurry Electrolyte**

Xin Liu ^1, 2#^, Zhang Chen ^1, 2#^, Daiqi Zhou ^1, 2^, Qi Zhao ^1, 2^, Haitao Feng ^3^, Yuanyuan Cui ^1, 2,^ *, Changsheng Ding ^1, 2,^ * and Yanfeng Gao ^1,2,^*

^1^ School of Materials Science and Engineering, Shanghai University, Shanghai 200444, P. R. China

^2^ State Key Laboratory of Advanced Refractories, Shanghai University, Shanghai 200444, P. R. China

^3^ Key Laboratory of Comprehensive and Highly Efficient Utilization of Salt Lake Resources, Qinghai Institute of Salt Lakes, Chinese Academy of Sciences, Xining 810008, P. R. China

#Xin Liu and Zhang Chen have contributed equally to this work.

*Corresponding authors. E-mail: [cui-yy@shu.edu.cn](mailto:cui-yy@shu.edu.cn) (Yuanyuan Cui); [dingcs@shu.edu.cn](mailto:dingcs@shu.edu.cn) (Changsheng Ding); [yfgao@shu.edu.cn](mailto:yfgao@shu.edu.cn) (Yanfeng Gao)

**Supplementary Figures**

**Fig. S1** Optical photographs of 0.5 M, 1.5 M, 2.5 M and 3.5 M MnO_2_ semi-solid slurry electrolytes.

**Fig. S2** Optical photographs of the MnO_2_ semi-solid electrolyte at different standing times.

**Fig. S3** The conductivity of slurry electrolytes with different concentrations of MnO_2_.

**Fig. S4** The charge–discharge curves of the batteries with 3.0 M MnO_2_ at the electrode area of 4 cm^2^.

**Fig. S5** Rate performance of the 1.0 M MnO_2_ semi-solid slurry electrolyte at the electrode area of 4 and 25 cm^2^.

**Fig. S6** Charge-discharge curves at 30 mA cm^-2^ under different MnO_2_ concentrations for a) 4 cm^2^ (areal capacity: 143.4 mAh cm^-2^) and b) 25 cm^2^ (areal capacity: 125 mAh cm^-2^) electrodes.

**Fig. S7** The 1^st^ and 2^nd^ charge–discharge curves of the semi-solid slurry electrolyte with 4 M MnO_2_ at the current density of 1 mA cm^-2^.

**Fig. S8** The charge-discharge curves at a current density of 50 mA cm^-2^.

**Fig. S9** Energy cost calculation of 4 M MnO_2_ semi-solid slurry electrolyte compared to other type RFBs.

**Fig. S10** Comparison of the current density and specific capacity for semi-solid RFBs.

**Fig. S11** Optical photographs of the semi-solid slurry electrolytes with different concentrations of MnO_2_ after 2^nd^ cycle.

**Fig. S12** The utilization rate of converting MnO_2_ to Mn^2+^ in semi-solid slurry electrolyte.

**Fig. S13** The viscosity measurements of the electrolyte after MnO_2_ dissolution into soluble Mn^2+^.

**Fig. S14** The concentrations of H⁺ consumed in the anolyte electrolyte after the 2^nd^ cycle at different MnO_2_ concentrations.

**Fig. S15** CV results of the semi-solid slurry electrolytes with a) 0.5 M, b) 1.0 M, c) 1.5 M, d) 2.0 M, e) 2.5 M, f) 3.0 M, g) 3.5 M and h) 4.0 M MnO_2_ after 2^nd^ cycle at various scan rates.

**Fig. S16** SEM images of commercial MnO_2_ deposited on graphite felt electrodes.

**Fig. S17** TEM images of MnO_2_ corresponding to Pot I and the corresponding magnified view (areas A and B).

**Fig. S18** The particle size distribution plots of a) commercial and b) electrolytic MnO_2_.

**Fig. S19** TEM image of electrolytic MnO_2_ corresponding to Pot Ⅴ.

**Fig. S20** Circuit fitting and charge transfer resistances of the commercial MnO_2_ and electrolytic MnO_2_.

**Fig. S21** a) CV result of the electrolytic MnO_2_ (Pot Ⅴ) at various scan rates and b) the relationship between oxidation and reduction peak current and scanning rate.

**Fig. S22** Optical photographs of 1 M commercial MnO_2_ and electrolytic MnO_2_ (Pot Ⅴ) semi-solid slurry electrolytes.

**Fig. S23** The charge-discharge curves for the electrolyte without Na_4_P_2_O_7_.

**Fig. S24** The specific capacity-voltage profiles of the 1.5 M MnO_2_ electrolyte at the electrode area of 4 cm^2^.

**Fig. S25** The specific capacity-voltage profiles of the a) 2.0 M MnO_2_, b) 3.0 M MnO_2_ and c) 4.0 M MnO_2_ electrolyte at the electrode area of 25 cm^2^.

**Fig. S26** Evolution of Mn and Fe concentrations in the catholyte and anolyte over 40 cycles using a 1.5 M MnO_2_ electrolyte with 4 cm^2^ electrode area.

**Fig. S27** The electrodes after long-term cycling at a) 1.0 M, b) 3.0 M and c) 4.0 M MnO_2_ with 25 cm^2^ area.

**Fig. S28** a) The capacity–voltage profiles and b) long-term cycling performance of Fe/Mn and V/Mn SSFBs.

**Table S1** The specific capacity, areal capacity and Mn species concentration in solution of semi-solid slurry electrolyte in this study.

|  | MnO_2_ concentration (4 cm^2^) | Specific Capacity (Ah L-1 Catholyte) | Areal  Capacity (mAh cm^-2^) | Mn species concentration |
| --- | --- | --- | --- | --- |
| 0.5 M | | 51.2 | 143.3 | 1.38 M |
|  | 1.0 M | 75.8 | 192.0 | 1.83 M |
|  | 1.5 M | 98.4 | 284.3 | 2.22 M |
| 2.0 M | | 109.7 | 369.0 | 2.58 M |
|  | 2.5 M | 121.8 | 411.4 | 2.99 M |
|  | MnO_2_ concentration (25 cm^2^) |  |  |  |
|  | 2.0 M | 111.6 | 89.3 | 2.62 M |
|  | 2.5 M | 120.2 | 96.2 | 2.97 M |
|  | 3.0 M | 134.9 | 107.9 | 3.25 M |
|  | 3.5 M | 147.7 | 118.1 | 3.49 M |
|  | 4.0 M | 156.2 | 125.0 | 3.76 M |

**Energy cost calculation (C_energy_):**

The chemical cost of storage can be calculated from equation (1): ^[31,32]^

$C_{\mathrm{energy}}= \frac{C_{\mathrm{chemical}} + C_{\mathrm{tank}}}{\varepsilon_{sys,d} \times\varepsilon_{q.rt}\times\varepsilon_{v,d}}$	(1)

The definitions of terms were list below:

| **Term** | **Definition** |
| --- | --- |
| **C_chemical_** | Combined cost of chemicals used in the negolyte and posolyte |
| **C_tank_** | Bulk tank price (0.15 US$ L^-1^) normalized by the energy density (kWh L^-1^) |
| **ε_sys,d_** | System round trip efficiency taken as a constant of 0.94 |
| **ε_q.rt_** | Coulombic efficiency of system |
| **ε_v.d_** | The discharge voltage divided by cell open circuit voltage |

All the references in supplementary information are also cited in main-text

Assuming a 1 Ah system, the chemical cost can be calculated based on equation. 2-4:^[31,32]^

$C_{\mathrm{chemical}}= \frac{Cost of negolyte + Cost of posolyte}{OCV \times1 \times0.001}$	(2)

Cost of negolyte (US$) $={[P}_{a}\times C_{a}+P_{a,s}\times C_{a,s}]\times\frac{1 Ah}{{CaP}_{a}}$	(3)

Cost of posolyte (US$) $={[P}_{c}\times C_{c}+P_{c,s}\times C_{c,s}]\times\frac{1 Ah}{{CaP}_{c}}$	(4)

The definitions of terms were list below:

| **Term** | **Definition** |
| --- | --- |
| ***P_a_* and *P_c_*** | Price (*𝑉𝑆$ mol^−1^*) of active materials |
| ***C_a_* and *C_c_*** | Concentration (*𝑛𝑜𝑚 𝐿^−1^*) of the active materials |
| ***P_a,s_* and *P_a,s_*** | Price (*𝑉𝑆$ mol^−1^*) of supporting materials |
| ***C_c,s_* and *C_c,s_*** | Concentration (*𝑛𝑜𝑚 𝐿^−1^*) of the supporting materials |
| ***Cap_a_* and *Cap_c_*** | Capacity (*Ah L^-1^*) at the given concentration |

All the references in supplementary information are also cited in main-text.

The bulk prices for chemicals are listed, and the chemical cost calculation details by using the above equations are presented in Table S3-S6.

**Table S2** Calculation of chemical cost of negolyte in this work

|  | Bulk price (US$ kg^-1^) | Concentration (mol L^-1^) | MW (kg mol^-1^) | Capacity (Ah L^-1^) | Cost (US$ Ah^-1^) |
| --- | --- | --- | --- | --- | --- |
| **Fe_2_(SO_4_)_3_** | 0.3 | 0.5 | 399.878 | 38 | 0.001578 |
| **FeSO_4_·7H_2_O** | 0.878 | 1 | 278.01 |  | 0.006423 |
| **MnSO_4_·H_2_O** | 0.35 | 1 | 169.01 |  | 0.001557 |
| **H_2_SO_4_** | 0.075 | 3 | 98 |  | 0.00058 |
| **Chemical Cost of Negolyte (US$ Ah^-1^)** | | | | | 0.010139 |

All the price from https://www.alibaba.com.

**Table S3** Calculation of chemical cost of posolyte in this work

|  | Bulk price (US$ kg^-1^) | Concentration (mol L^-1^) | MW (kg mol^-1^) | Capacity (Ah L^-1^) | Cost (US$ Ah^-1^) |
| --- | --- | --- | --- | --- | --- |
| **MnSO_4_·H_2_O** | 0.35 | 1 | 169.01 | 156.2 | 0.000379 |
| **Fe_2_(SO_4_)_3_** | 0.3 | 0.5 | 399.878 |  | 0.000384 |
| **Na_4_P_2_O_7_·10H_2_O** | 7.027 | 0.1 | 446.05 |  | 0.002007 |
| **MnO_2_** | 1.5 | 4 | 86.937 |  | 0.003339 |
| **H_2_SO_4_** | 0.075 | 3 | 98 |  | 0.000141 |
| **Chemical Cost of Posolyte (US$ Ah^-1^)** | | | | | 0.00625 |

All the price from https://www.alibaba.com.

**Table S4** Calculation of chemical cost of negolyte and posolyte in Ref. 21

|  | Bulk price (US$ kg^-1^) | Concentration (mol L^-1^) | MW (kg mol^-1^) | Capacity (Ah L^-1^) | Cost (US$ Ah^-1^) |
| --- | --- | --- | --- | --- | --- |
| **MnBr_2_** | 10.5 | 2 | 214.746 | 200 | 0.022548 |
| **MnSO_4_·H_2_O** | 0.35 | 1 | 169.01 |  | 0.000296 |
| **HClO_4_** | 98.385 | 2 | 100.46 |  | 0.098838 |
| **Chemical Cost (US$ Ah^-1^)** | | | | | 0.121682 |

All the price from https://www.alibaba.com.

**Table S5** Calculation of energy cost

|  | **This work** | **Ref.21** |
| --- | --- | --- |
| **Cost of negolyte (US$ Ah^-1^)** | 0.00625 | 0.121682 |
| **Cost of posolyte (US$ Ah^-1^)** | 0.010139 | 0.121682 |
| **OCV (V)** | 0.79 | 2 |
| **C_chemical_ (US$ kWh^-1^)** | 20.74557 | 121.682 |
| **C_tank_ (US$ kWh^-1^)** | 6.212217 | 0.83333 |
| **ε_sys,d_** | 0.94 | 0.94 |
| **ε_q.rt_** | 0.99 | 0.99 |
| **ε_v.d_** | 0.81 | 0.76 |
| **C_energy_ (US$ kWh^-1^)** | 35.76318 | 173.2263 |

**Table S6** The comparison of specific capacity, areal capacity, current density and energy cost for reported Mn-based RFBs

|  | Battery Type | Specific Capacity (Ah L-1 Catholyte) | Areal  Capacity (mAh cm^-2^) | Current Density (mA cm^-2^) | Energy cost (US$ kWh^-1^) | Ref |
| --- | --- | --- | --- | --- | --- | --- |
| Zn-Mn | | 18 | 40 | 20 | / | 13 |
|  | Fe-Mn | 36 | 142 | 50 | 53 | 15 |
|  | Cd-Mn | 200 | 61 | 10 | 173 | 21 |
| Zn-Mn | | 105 | / | 30 | / | 22 |
|  | Zn-Mn semi-solid slurry | 92 | / | 1 | 48 | 29 |
|  | Zn-Mn semi-solid slurry | 25 | / | 0.2 | / | 30 |
|  | Zn-Mn | 30 | 20 | 40 | / | 34 |
|  | S-Mn | 40 | 100 | 10 | / | 35 |
|  | Zn-Mn | 25 | 50 | 20 | / | 36 |
|  | Zn-Mn | 40 | 80 | 20 | / | 37 |
|  | Fe-Mn semi-solid slurry | 156 | 125 | 30 | 36 | This work |
|  |  | 125 | 100 | 40 |  |  |
|  |  | 183 | 146 | 1 |  |  |

All the references in supplementary information are also cited in main-text.

**Table S7** The comparison of specific capacity and current density for reported semi-solid RFBs

|  | Semi-solid electrolyte | Specific Capacity (Ah L^-1^) | Current Density (mA cm^-2^) | Ref |
| --- | --- | --- | --- | --- |
|  | 11 vol% MnO_2_ + 4.2 vol% CB | 92 | 1 | 29 |
|  | Mn_3_O_4_@C-MWCNTs | 25 | 0.2 | 30 |
| 10 wt% SiO_x_ | | 98 | 0.2 | 38 |
|  | 10 wt% SiO_x_@LiPN/S | 207 | 0.2 | 39 |
|  | 5 vol% S-HCS | 82.6 | 1 | 40 |
| 24 vol% CSP@C-LiPAA | | 280 | 0.3 | 41 |
|  | 40 vol% LPF-CF | 68 | 0.1 | 42 |
|  | 20 vol% LFP-PVP | 29 | 1.67 | 43 |
|  | 138 g L^-1^ S-KB@rGO | 139 | 0.5 | 44 |
|  | 150 g L^-1^ FeSe_2_ | 36.4 | 24 | 45 |
|  | Fe_3_O_4_ | 99.2 | 20 | 46 |
|  | Polyoxometalate colloid | 6.3 | 20 | 47 |
|  | 4 M MnO_2_ | 156 | 30 | This work |
|  |  | 125 | 40 |  |
|  |  | 183 | 1 |  |

All the references in supplementary information are also cited in main-text.

**Supplementary Note S1**

In this work, MnSO_4_ and Fe_2_(SO_4_)_3_ were selected as the cathode and anode soluble active species of the battery, respectively, and their respective redox reactions during 1^st^ charging are as follows:

Cathode: Mn^3+^ + e^-^ ↔ Mn^2+^ (1.56 V vs SHE)

Anode: Fe^2+^ ↔ Fe^3+^ + e^-^ (0.77 V vs SHE)

Adding MnO_2_ solid particles to the pristine electrolyte requires extra FeSO_4_ in the anolyte to match the MnO_2_ electrochemical reaction. The electrochemical reactions occurring during the 1^st^ discharge are presented below.

Cathode: MnO_2_ + 4H^+^ + 2e^-^ → Mn^2+^ + 2H_2_O (1.22 V vs SHE)

Anode: Fe^2+^ - e^-^ ↔ Fe^3+^ (0.77 V vs SHE)

According to the electrochemical equations, 2 M of Fe^2+^ are required for the complete reaction of 1 M MnO_2_. Given the low solubility of FeSO_4_ (≤ 1 M), a larger anolyte volume is necessary primarily to supply sufficient Fe^2+^ for the reaction, with proton provision serving as an ancillary function. A reduction in anolyte volume lowers the total Fe^2+^ content, thereby reducing the amount of MnO_2_ that can react and the corresponding proton demand for charge balance.
